# Establishment and Characterization of Paired Primary Cultures of Human Pancreatic Cancer Cells and Stellate Cells Derived from the Same Tumor

**DOI:** 10.3390/cells9010227

**Published:** 2020-01-16

**Authors:** Manoj Amrutkar, Emma Kristine Larsen, Monica Aasrum, Anette Vefferstad Finstadsveen, Per Arne Andresen, Caroline S. Verbeke, Ivar P. Gladhaug

**Affiliations:** 1 Department of Pharmacology, Institute of Clinical Medicine, University of Oslo, Blindern, 0316 Oslo, Norway; emma@me.online.no (E.K.L.); monica.aasrum@medisin.uio.no (M.A.); 2 Department of Hepato-Pancreato-Biliary Surgery, Institute of Clinical Medicine, University of Oslo, Blindern, 0318 Oslo, Norway; i.p.gladhaug@medisin.uio.no; 3 Department of Pathology, Oslo University Hospital, Rikshospitalet, Nydalen, 0424 Oslo, Norway; uxvene@ous-hf.no (A.V.F.); pandrese@ous-hf.no (P.A.A.); c.s.verbeke@medisin.uio.no (C.S.V.); 4 Department of Pathology, Institute of Clinical Medicine, University of Oslo, Blindern, 0316 Oslo, Norway; 5 Department of Hepato-Pancreato-Biliary Surgery, Oslo University Hospital, Rikshospitalet, Nydalen, 0424 Oslo, Norway

**Keywords:** human pancreatic ductal adenocarcinoma, primary cultures, pancreatic stellate cells, and cancer cells, secretome

## Abstract

Pancreatic ductal adenocarcinoma (PDAC) is characterized by an extremely poor prognosis, and its treatment remains a challenge. As the existing in vitro experimental models offer only a limited resemblance to human PDAC, there is a strong need for additional research tools to better understand PDAC tumor biology, particularly the impact of the tumor stroma. Here, we report for the first time the establishment and characterization of human PDAC-derived paired primary monolayer cultures of (epithelial) cancer cells (PCCs) and mesenchymal stellate cells (PSCs) derived from the same tumor by the outgrowth method. Characterization of cell morphology, cytostructural, and functional profiles and proteomics-based secretome analysis were performed. All PCCs harbored *KRAS* and *TP53* mutations, and expressed cytokeratin 19, ki-67, and p53, while the expression of EpCAM and vimentin was variable. All PSCs expressed α-smooth muscle actin (α-SMA) and vimentin. PCCs showed a significantly higher growth rate and proliferation than PSCs. Secretome analysis confirmed the distinct nature of PCCs as compared to PSCs and allowed identification of potential molecular regulators of PSC-conditioned medium (PSC-CM)-induced migration of PCCs. Paired primary cultures of PCCs and PSCs derived from the same tumor specimen represent a novel experimental model for basic research in PDAC tumor biology.

## 1. Introduction

Pancreatic ductal adenocarcinoma (PDAC), commonly known as pancreatic cancer, is one of the most lethal solid tumors, characterized by early metastasis, a complex tumor microenvironment, profound chemoresistance, and overall 5-year survival of less than 7% [1,2]. Despite new insight in the genomic basis of the disease, therapeutic advancement for PDAC has been negligible over the past decades [3,4,5]. Presently, PDAC is the fourth most common cause of cancer deaths in the Western world and is expected to rank second by 2030 [6]. Globally, about 459,000 new cases of pancreatic cancer have been reported in 2018, and the incidence is projected to rise significantly during next decade [6,7,8]. Only 15–20% of all PDAC patients are eligible for surgery with curable intent; however, a resectable disease also carries a high risk of disease recurrence within the first-year post-surgery, implying a critical need for additional treatment modalities [9]. The profound resistance of PDAC to conventional chemotherapy is considered a major impediment to improved survival [10].

The tumor microenvironment (TME) in PDAC is unique because of its prominent stroma containing activated pancreatic stellate cells (PSCs; also referred to as cancer-associated fibroblasts, CAFs) as the major cellular component [11,12,13]. PSCs produce excessive amounts of extracellular matrix (ECM) components such as collagen, fibronectin, laminin, and hyaluronic acid, which altogether create a dense biophysical structure surrounding the malignant epithelial pancreatic cancer cells (PCCs) [14,15]. The continuous deposition of ECM leads to a hypoxic and nutrient-poor microenvironment that impairs drug delivery and induces resistance to chemotherapeutic agents in carcinoma cells [11,16]. Several lines of evidence point to the importance of bidirectional interactions between PCCs and PSCs as the underlying mechanisms in tumor invasion, metastasis, and chemoresistance [17,18,19]. Hence, one possible explanation for the lack of improvement in PDAC prognosis is that existing and experimental PDAC therapies typically utilize cancer cell-centric approaches and have failed to recognize PDAC as a complex biologic system of pancreatic cancer cells; stromal cells, particularly PSCs; and ECM components [17,20].

In the past, basic research within the pancreatic cancer field has been mainly performed by employing monocultures of a set of widely available commercial cancer cell lines [21]. However, these have certain disadvantages, particularly regarding their representativeness of the original tumor, because human PDACs exhibit profound intratumor heterogeneity. Furthermore, some cell lines are derived from metastases and all are prone to genetic drift. In recent years, various primary pancreatic carcinoma cell lines have been established in an attempt to overcome these limitations [22,23,24]. However, these models lack the complex input from the stromal components found in all PDACs, particularly cues from the PSCs. Similar to cancer cell lines, research on PSCs has mainly been conducted using a few commercially available PSC lines, which differ phenotypically and in their interactions with cancer cells compared to primary established PSCs, as shown in our recent study [25]. Several recent reports on various 2D and 3D models attempting to mimic the microenvironment of PDACs have invariably used PCC and PSC combinations of primary cells and immortalized cells obtained from different tumors [26,27,28,29,30]. This prompted us to establish an experimental model of selective pairs of primary PCCs and primary PSCs obtained from the same surgically resected human PDAC tumor.

Here, we report the establishment of a novel model system of human PDAC-derived paired primary cultures of pancreatic cancer cells and stellate cells isolated from six primary pancreatic cancers. The primary cells were investigated for genetic, immunohistochemical, and functional characteristics as well as the composition of their secretome. Furthermore, we exploited the model to study the effect of the PSC secretome on the proliferation and migration of the paired PCCs. 

## 2. Materials and Methods

### 2.1. Patients

The study protocol and patient consent documents were approved by the Regional Committee for Medical and Health Research Ethics (REC South East, project number 2015/738) and followed the Helsinki Declaration. Written informed consent was obtained from the patients enrolled in the study.

### 2.2. Reagents

Reagents were purchased from the following sources: Dulbecco’s modified Eagle’s medium (DMEM) containing 4.5 g/L glucose, penicillin-streptomycin (Pen-Strep), Amphotericin B, Trypsin/EDTA, fetal bovine serum (FBS), and Pierce^TM^ BCA protein assay kit from Thermo Fisher Scientific (Waltham, MA, USA); bovine serum albumin (BSA), 3-(4,5-Dimethylthiazol-2-yl)-2,5-Diphenyltetrazolium Bromide (MTT), and phosphate-buffered saline (PBS) from Sigma-Aldrich (St Louis, MO, USA); and Ultima Gold from Perkin Elmer (Waltham, MA, USA).

### 2.3. Cell Culture

Human PDAC-derived paired primary cultures of pancreatic cancer cells (PCCs) and stellate cells (PSCs) were obtained from the same surgically resected tumor from six different PDAC patients (PC-1, PC-2, PC-3, PC-4, PC-5, and PC-6) using the outgrowth method [14,25] as schematically presented in Supplementary Materials Figure S1. Of these, PC-1, PC-2, and PC-3 were treatment naïve (TN), while PC-4, PC-5, and PC-6 had undergone neoadjuvant treatment (NAT). Clinicopathological features of the tumors, patient survival, and treatment information are provided in Table 1. The established cancer and stromal cell cultures were designated as PCC-1, -2, -3, -4, -5, and -6 and as PSC-1, -2, -3, -4, -5, and -6, respectively. Of note, the PSCs from PC-1 were not sufficient to undertake further experiments; hence, they were not included in the study. Based on the treatment status of source PDACs, the cultures are referred as TN-PCCs, TN-PSCs, NAT-PCCs, and NAT-PSCs. The following controls were used in this study: the pancreatic cancer cell lines AsPC-1, BxPC-3, and Panc-1 (ATCC, Manassas, VA, USA) and HPaSteC (Human Pancreatic Stellate Cells, ScienCell Research Laboratories (San Diego, CA, USA). Both PCC and PSC cultures (harvested between passage 3 and 8) and the control cell lines were cultured and maintained in DMEM supplemented with 10% FBS, 1% Pen-Strep, and 1% Amphotericin B. 

### 2.4. Morphology and Immunocytochemistry

PDAC tissues were fixed in 10% neutral buffered formalin for 24 h and transferred to 70% ethanol. Tissues were embedded in paraffin, and 3–5 μm sections were processed for hematoxylin and eosin (H&E) staining using standard protocols as previously described [31]. H&E stained sections were used for the assessment of clinicopathological features of the tumor. 

Cells cultured in 96-well plates were fixed in 4% formaldehyde, followed by morphology assessment or immunocytochemistry as described previously [25]. Briefly, the cells were stained with hematoxylin and eosin (H&E); images were captured under the light microscope and assessed for morphology. For immunocytochemistry, cells were incubated overnight with various primary antibodies, followed by staining with Alexa Fluor-conjugated secondary antibodies. DAPI was used for nuclear staining. Images were captured using EVOS FLoid Cell Imaging Station (Thermo Fisher Scientific). Antibody details are provided in Supplementary Materials Table S1. 

### 2.5. Western Blot Analysis

Whole cell lysates were prepared using Laemmli buffer, and aliquots of protein were separated on 10% polyacrylamide gels by electrophoresis (SDS-PAGE), as described previously [19,25]. The proteins were transferred to nitrocellulose membranes using a semi-dry transfer system (Bio-Rad, Hercules, CA, USA), blocked in 5% non-fat dry milk solution, and incubated overnight at 4 °C with the primary antibodies as indicated. The next day, blots were incubated with HRP-conjugated secondary antibodies at room temperature for 1 h and visualized with LumiGLO^®^ (KPL, Gaithersburg, MD, USA). The densitometric analysis was performed using Labworks Software (UVP, Cambridge, UK). Antibody details are provided in Supplementary Materials Table S1.

### 2.6. Mutational Analysis

Genomic DNA was isolated from six PCC cultures using DNeasy Blood & Tissue Kit (Qiagen) in accordance with the manufacturer’s protocol. DNA yield and purity were assessed using the Qubit 3.0 Fluorometer (Thermo Fisher Scientific). The samples were subjected to next-generation sequencing (NGS) on an in-house designed 360 gene panel at the Mohn Cancer Research Laboratory, University of Bergen, Norway [32]. The panel targets gene markers were associated with cancer development in compliance with the census of cancer genes cataloged in the COSMIC database and genes recurrently overexpressed in cancer [33,34]. Information on genetic differences among the cancer cell lines was extracted for six genes (*KRAS*, *CDKN2A*, *TP53*, *SMAD4*, *BRCA1,* and *BRCA2*). Allele fraction (AF) values were used as indicators of homo- or heterozygous representation of the genetic variants disclosed. To select for somatically induced alterations, variants identified more than once in any of the reference population-based data sets (e.g., 1000 g) and less than twice in the COSMIC database were deemed as germline and excluded from further assessment. Categorization into damaging or tolerant mutations was guided by in silico models incorporated into the annotation algorithms.

### 2.7. Cell Growth and Proliferation

For measurement of growth curves and doubling time, approximately 20,000 PCCs and 3000 PSCs per well in 24- and 96-well plates, respectively, were cultured and maintained in complete growth medium. The number of viable cells was counted every 24 h over a 4-day period. Cell population doubling time was calculated from the logarithmic growth curves, using the formula described previously [25]. To determine the proliferation rate, the MTT-based cell viability assay was performed. Briefly, 3000 cells per well were seeded in a 96-well plate, and the change in the number of viable cells over a 48 h period was calculated, as described previously [25]. The proliferation rate was calculated as the percentage change in the number of viable cells relative to the time interval.

### 2.8. Preparation of Conditioned Media

PCC- and PSC-conditioned medium, designated as PCC-CM and PSC-CM, respectively, was obtained, as described previously [25]. Briefly, sub-confluent individual cultures of PCCs and PSCs in 10-cm^2^ petri dishes were washed thoroughly with PBS and incubated with fresh serum-free DMEM (SFM; ~10 mL) for 48 h. The culture supernatants (i.e., the conditioned medium) was collected, centrifuged, and stored at –20 °C until further use. 

### 2.9. Secretome Analysis

The proteomics-based secretome analysis of the PCC-CM and PSC-CM was performed using mass spectrometry (MS) analysis, as described in our recent study [25]. The workflow of the procedure is described in Supplementary Materials Figure S2A. Briefly, conditioned medium obtained from six PCC and five PSC cultures was subjected to proteomics-based MS analysis. Conditioned medium from the pancreatic cancer cell lines AsPC-1, BxPC-3, and Panc-1 and human pancreatic stellate cells HPaSteC was used as positive controls. The conditioned media preparations were concentrated down to 5% of their original volume, followed by overnight protein digestion at 37 °C. Prior to MS; the resulting peptides were desalted and concentrated by the STAGE-TIP method. Each peptide mixture was analyzed by nEASY-LC coupled to QExactive Plus (ThermoElectron, Bremen, Germany) with EASY Spray PepMap^®^RSLC column. The resulting MS raw files were analysed with MaxQuant software version 1.6.2.10 for protein identification and label-free quantification using the Andromeda search engine, following the settings as described previously [25]. The databased search was performed using the SwissProt human database. Perseus software, version 1.5.6.0, was used for statistical analysis. Pathway analysis of identified proteins was performed using the Kyoto Encyclopedia of Genes and Genomes (KEGG) database [35]. Gene ontology (GO) analysis was also conducted using the functional annotation tool that is made available through the DAVID Bioinformatics Database version 6.7 [36,37]. 

### 2.10. Effect of PSC-CM on Proliferation and Migration of PCCs

To assess the effect of PSC-CM on the proliferation and migration capacity of PCCs, the [^3^H]-thymidine incorporation assay for DNA synthesis measurement [38] and the scratch assay to study the migration of cancer cells [18] were employed. For measurement of DNA synthesis, PCCs seeded in 12-well plates were stimulated with their paired PSC-CM or SFM for 24 h and pulsed with [^3^H]-thymidine (1 μCi/mL) for the last 4 h of stimulation, following which the incorporation of [^3^H]-thymidine was determined by liquid scintillation counting. To study cell migration, scratch wounds were made in confluent PCCs seeded in 12-well plates, followed by incubation with SFM or PSC-CM for 24 h. After the addition of SFM or PSC-CM, images were taken immediately, i.e., at 0 h, and at 24 h with an Olympus F-View II Soft Imaging System High-Resolution CCD Camera. For each picture, the wound area was measured by FIJI software, and the percent wound closure was calculated as previously described [39].

### 2.11. Statistical Analysis

All values are expressed as mean ± standard error of the mean (SEM). Statistical analysis was performed using GraphPad Prism 6 Software. For comparison between two groups, an unpaired two-tailed Student’s t-test was used. A value of *p* < 0.05 was considered statistically significant.

## 3. Results

### 3.1. Clinical Summary of Patients

**Figure 1:**
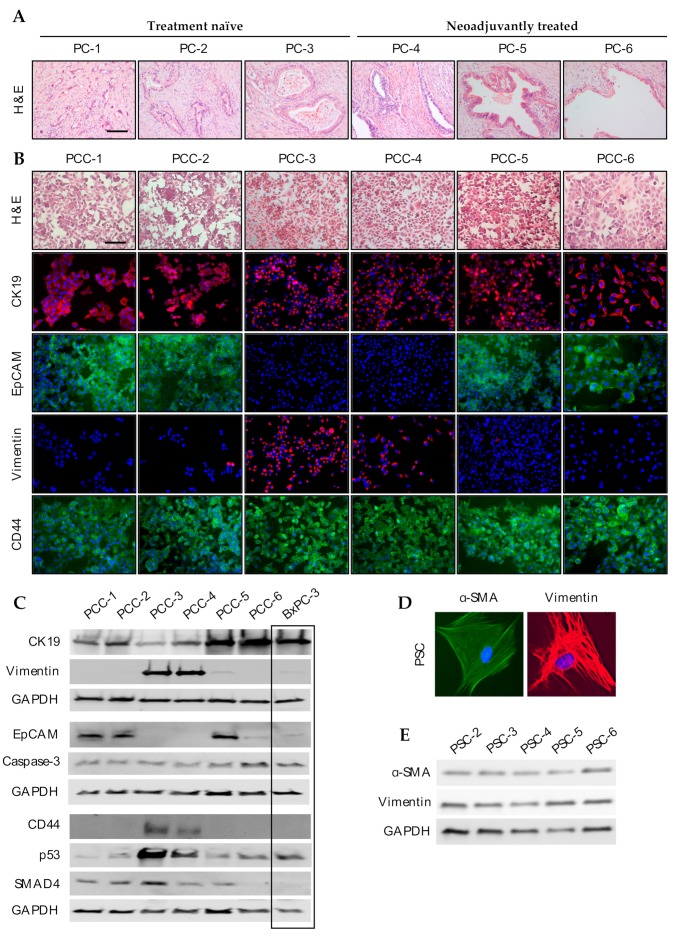
Morphology and expression analysis: (**A**) H&E staining of representative source tumors from which PCCs and PSCs were derived. Scala bar = 50 µm. Paired primary cultures of PCCs and PSCs originated from the same individual PDAC tumor specimen assessed for morphology and expression analysis: (**B**) PCCs stained with hematoxylin and eosin (H&E); immunostained with antibodies against cytokeratin 19 (CK19), transmembrane glycoprotein epithelial cell adhesion molecule (EpCAM), vimentin, and CD44. Scale bar = 100 µm. (**C**) PCCs were lysed and proteins were subjected to immunoblotting using antibodies against CK19, EpCAM, vimentin, CD44, p53, SMAD4, and Caspase-3. (**D**) PSCs immunostained with anti- α-smooth muscle actin (α-SMA; green) and anti-vimentin (red) antibodies. (**E**) PSCs were lysed and proteins were subjected to immunoblotting using antibodies against α-SMA and vimentin. For Figure 1B,D, nuclei were stained with DAPI (blue). GAPDH was used as a loading control for Figure 1C,E. PC, pancreatic cancer; PCC, pancreatic cancer cell; PSC, pancreatic stellate cell.

Histopathological evaluation of H&E-stained sections of the source tumors (PC-1 to PC-6) from which PCCs and PSCs originated confirmed ductal adenocarcinoma in all six patients (Figure 1A). The latter three PDACs in the panel, PC-4, PC-5, and PC-6, were neoadjuvantly treated and displayed poor response to the treatment, as indicated by their tumor regression grades of 2–3 (Table 1). Except for PC-4, the post-surgery survival of patients was shorter than two years. For additional details, see Table 1. 

### 3.2. Outgrowth Efficiency

From tumors of the 51 patients included in this study, we were able to isolate six paired primary cultures of PCCs and PSCs. This represents an outgrowth success rate of 11.8% for paired cultures. The successful outgrowth of PSCs alone was obtained from 33 tumor specimens, indicating 64.7% outgrowth efficiency for PSCs. Initial tumor outgrowth also occurred in the same number of specimens as outgrowth of PSCs; however, further culturing was not successful. The major obstacle to establish viable paired cultures of PCCs and PSCs from all specimens were senescence, and minor reasons were infected cultures (fungal infection in three cases and bacterial contamination in two cases).

### 3.3. Phenotypic Characterization of PCCs and PSCs

All PCCs and PSCs grew as an adherent monolayer. Morphological assessment of H&E-stained PCCs revealed that all six cultures were composed of polygonal-shaped cells with ovoid nuclei and exhibited an epithelial growth pattern (Figure 1B). The cultures PCC-1, -2, -5, and -6 were homogenous in size, whereas PCC-3 and -4 were heterogeneous such that the cultures consisted of both small and large cells with the presence of a few elongated cells. PCC-1 and -6 were relatively large-sized, whereas PCC-2 and -5 were relatively smaller. Next, the expression of various cancer cell-associated marker proteins was investigated by immunocytochemistry and western blot analysis (Figure 1B; Supplementary Materials Figure S3). All PCC cultures showed expression of the epithelial marker cytokeratin 19 (CK19), however, with a variable staining pattern, cytoplasmic among PCC-1, -2, -5, and -6 and perinuclear in PCC-3 and -4 (Figure 1B,C). Furthermore, heterogeneous expression of the transmembrane glycoprotein epithelial cell adhesion molecule (EpCAM) and the mesenchymal marker vimentin was observed among the PCC cultures. PCC-1, -2, -5, and -6 showed expression of EpCAM and were mostly negative for vimentin, whereas an opposite pattern was observed in PCC-3 and -4 (Figure 1B,C). Positive staining of the cancer stem cell marker CD44 (Figure 1B), the apoptosis marker Caspase-3, and the tumor suppressor p16 (Supplementary Materials Figure S3) was observed across all PCC cultures. Surprisingly, CD44 expression was not detectable in four of the PCCs by western blot analysis, although it was detected in all PCCs using immunostaining (Figure 1B,C). PCC-3 and -4 showed higher p53 expression compared to other PCCs in the panel, whereas SMAD4 expression was detected in all PCCs except PCC-6 (Figure 1C). A summary of the expression results is provided in Table 2. 

Genomic DNA subjected to exome sequencing revealed the presence of activating mutations *KRAS* in all PCC cultures. Allele fraction (AF) values indicated homozygosity for the *KRAS* mutations in PCC-3 and -4 (AF-values: 1.0). Three of the cell lines tested positive for a distinct *TP53* mutation of which the mutations in cell lines PCC-1 and -6 were predicted to be incapacitating (“damaging”) whereas the third in PCC-4 was of unknown significance. Loss of heterozygosity (*TP53*-LOH) was indicated by mutated allele fraction assessment in PCC-6 (AF = 1.0). Two mutations altering the reading frames of *CDKN2A* affecting both p16^INK4a^ and p14^ARF^ were identified in PCC-1, -2, and -5. No mutations were detected in *SMAD4* or *BRCA1*, but a *BRCA2* frameshift mutation was disclosed in PCC-1, -2, and -5. For additional details, see Table 3. 

In PSCs, immunostaining demonstrated a strong expression of vimentin and the activation marker α-smooth muscle actin (α-SMA) (Figure 1D), which was also confirmed by western blot analysis of whole cell lysates (Figure 1E), in accordance with our previous observations [19,25]. None of the PSC cultures expressed EpCAM (data not shown), excluding the possibility of contamination by epithelial cells during isolation. Glial fibrillary acidic protein (GFAP) was not detected in any of the PSC cultures (data not shown), which is in accordance with the reported loss during culturing [40]. 

### 3.4. Assessment of Cell Growth and Proliferation

**Figure 2:**
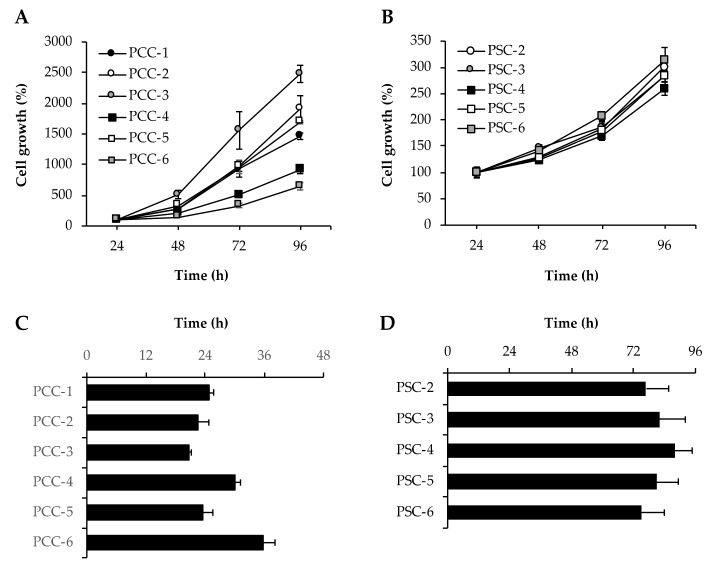
Assessment of growth: PCCs and PSCs seeded in 96-well plates individually were counted for cell number every 24 h for 4 days to determine growth curves (**A**,**B**) and doubling time (**C**,**D**). Data are mean ± SEM of triplicate determinations. PCC, pancreatic cancer cell; PSC, pancreatic stellate cell.

Growth curves and doubling times of both PCCs and PSCs were determined by cell counting at 24-h intervals over a 4-day period. PCCs were growing at a significantly faster rate than PSCs (Figure 2A,B). Among the PCCs, treatment-naïve PCC-3 was growing fastest, whereas neoadjuvantly treated PCC-6 was the slowest at all time points (Figure 2A). Doubling times as determined from the growth curves were lowest for PCC-3 (20.8 ± 0.5 h) and highest for PCC-6 (36.0 ± 2.5 h) (Figure 2C). The growth pattern of all PSC cultures was identical (Figure 2B). No significant differences were observed between the doubling times of the various PSC cultures (Figure 2D). Notably, the average doubling time for PSCs (80.6 ± 8.6 h) was >3-fold higher compared to PCCs (26.3 ± 1.5 h; Figure 2C,D). 

**Figure 3:**
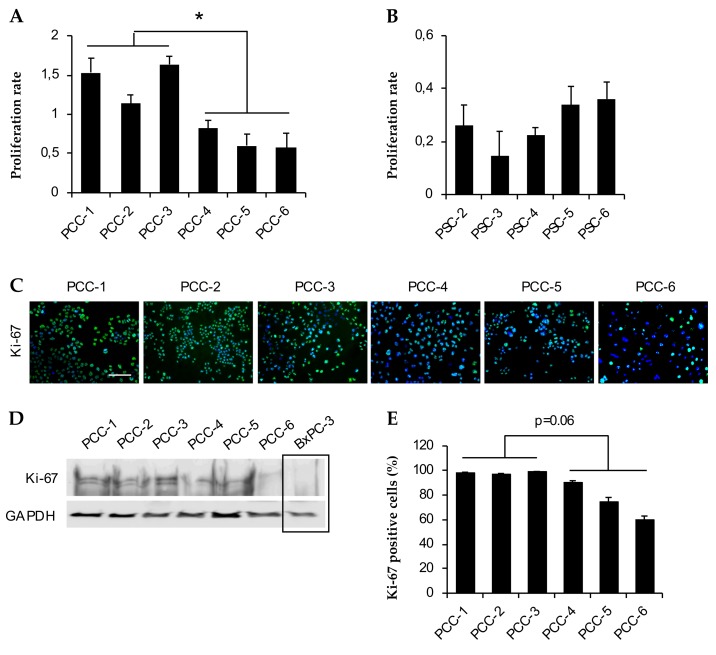
Assessment of proliferation: Cell proliferation rate obtained by measuring the percentage cell viability for PCCs (**A**) and PSCs (**B**) using 3-(4,5-Dimethylthiazol-2-yl)-2,5-Diphenyltetrazolium Bromide (MTT) assay at 24 and 72 h after cell seeding. * *p* < 0.05 comparing average proliferation rate for PCCs between treatment naïve (PCC-1, -2, and -3) vs neoadjuvantly treated (PCC-4, -5, and -6) group. (**C**) PCCs immunostained with antibodies against Ki-67 (green). Nuclei were stained with DAPI (blue). Scale bar = 100 µm. (**D**) PCCs were lysed and proteins were subjected to immunoblotting using antibodies against Ki-67. GAPDH was used as a loading control. (**E**) Cells seeded in 96-well plates were immunostained with antibodies against Ki-67. Ten randomly selected fields (20× magnification) from each well were counted to determine the percentage positive Ki-67 cells. Data are mean ± SEM of triplicate determinations. PCC, pancreatic cancer cell; PSC, pancreatic stellate cell.

The data on growth curves and doubling times were further corroborated by assessment of the proliferation rate based on MTT cell viability assay (Figure 3A,B). Among the PCCs, PCC-3 and PCC-6 displayed the highest and lowest proliferation rates, respectively. Notably, the average proliferation rate of TN-PCCs was significantly higher than that of NAT-PCCs (2-fold, *p* = 0.01; Figure 3A). Among the PSCs, PSC-3 and -6 displayed the lowest and highest proliferation rates, respectively (Figure 3B). Immunostaining with antibodies against Ki-67 showed variable expression across all PCC cultures (Figure 3C,D). The percentage Ki-67 positive cells ranged between 96–99% for PCC-1, -2, and -3, whereas it was 91, 74, and 60% for PCC-4, -5, and -6, respectively (Figure 3E). 

### 3.5. Proteomics Profiling of PCC- and PSC-Conditioned Medium

**Figure 4:**
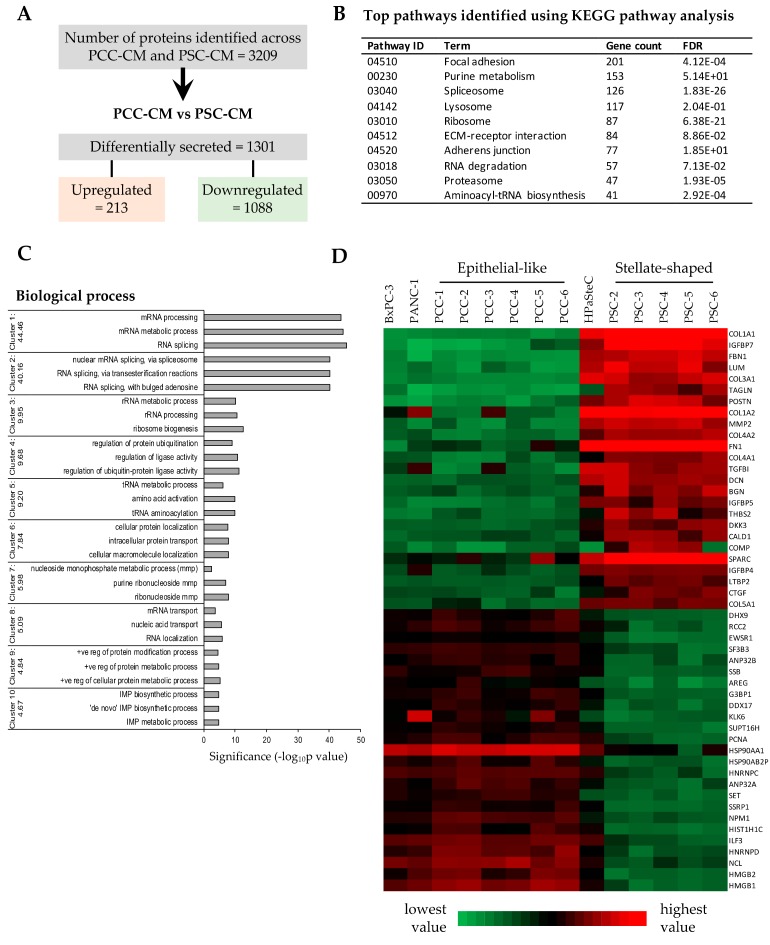
Proteome profiling of PCC-CM and PSC-CM preparations: Conditioned medium from six different PCC and five PSC cultures were subjected to proteomics analysis using LC-MS/MS. (**A**) Number of proteins detected, differentially secreted by PCCs and PSCs. (**B**) Most enriched Kyoto Encyclopedia of Genes and Genomes (KEGG) pathway identified. (**C**) DAVID Bioinformatics Resource tool based functional annotation of differentially secreted proteins by PCCs and PSCs. The representative Gene Ontology (GO) cluster groups with top 10 enrichment score are presented. The horizontal axis represents the significance (*p*-value) for each term, while the vertical axis represents the GO categories. (**D**) Heatmap of protein abundance pattern for the 50 most significantly downregulated and upregulated proteins. Red and green color indicates high and low expression, respectively. GO, Gene Ontology; PCC, pancreatic cancer cell; PSC, pancreatic stellate cell; HPaSteC, PSCs from normal human pancreas; PCC-CM or PSC-CM, conditioned medium from PCCs or PSCs.

The composition of conditioned medium from various PCC and PSC cultures and control BxPC-3, Panc-1, and HPaSteC cells was investigated by proteomics-based MS analysis that identified a maximum of 3209 different proteins (*Homo sapiens*) (Figure 4A). A complete list of all proteins together with their identification parameters is provided in Supplementary Materials Table S2. Expression levels of proteins secreted by PCCs and PSCs were comparable to those of the respective controls, confirming the quality and purity of cultures. The secretome sample subjected to MS analysis from ASPC-1 cells was an outlier as seen in the PCA plot (Supplementary Materials Table S2) and was therefore removed from further analysis. When comparing the secretome preparations of PCCs and PSCs, a total of 1301, i.e., 40.5% of the total proteins identified, was differentially regulated (Figure 4A, Supplementary Materials Table S3). A majority of these differentially secreted proteins were downregulated (1088, i.e., 83.6%), whereas the remainder were upregulated (Supplementary Materials Table S3). 

The list of differentially secreted proteins by the various PCC and PSC cultures was subjected to KEGG pathway analysis. A list of the top 10 enriched KEGG pathways is provided in Figure 4B. To determine the functional role of the differentially secreted proteins, a GO analysis was undertaken using the DAVID bioinformatic resource tool version 6.7 that revealed a significant representation of biological process categories that are mainly related to RNA and protein processing (Figure 4C). A list of the 50 most differentially regulated proteins, comprising the 25 each most down- and upregulated proteins, was selected for further analysis. A heatmap of these 50 most differentially regulated proteins revealed that PSCs secrete significantly larger amounts of major ECM components compared to PCCs (Figure 4D, Supplementary Materials Table S3). Notably, the levels of collagens COL1A1 and COL1A2, fibronectin, and SPARC were relatively higher than other ECM components (Figure 4D, Supplementary Materials Table S3). Interestingly PCCs displayed significantly higher expression of Heat shock protein HSP 90-alpha (HSP90AA1) (Figure 4D, Supplementary Materials Table S3). STRING-based analysis and GO analysis revealed that the majority of the most differentially secreted proteins belong to structural cellular components such as ECM; intracellular and membrane-bound organelles; or biological processes such as ECM organization, regulation of cellular metabolic processes, cell growth and communication, and transcription- and translation-related processes (Supplementary Materials Figure S2B and Table S3). 

Compared to TN-PCCs, the secretome of NAT-PCCs contained a total of 15 differentially regulated proteins, of which ten and five were up- and downregulated, respectively. Similarly, compared to TN-PSCs, the NAT-PSC secretome included a total of 37 differentially regulated proteins, of which three and 34 were up- and downregulated, respectively. A heatmap of these proteins along with their known functions is provided in Supplementary Materials Figure S4A,B. 

### 3.6. Effects of PSC-Conditioned Medium on DNA Synthesis and Migration of Paired PCCs

**Figure 5:**
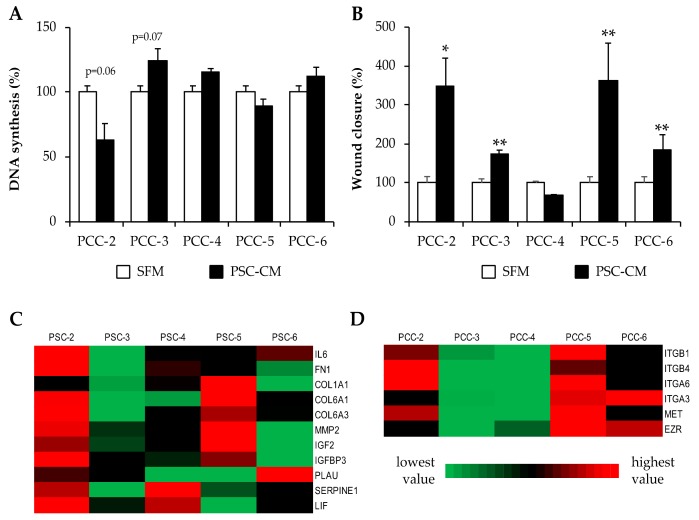
Effect of PSC-CM on proliferation and migration of paired PCCs: (**A**) PCCs incubated for 24 h with SFM or paired PSC-CM and DNA synthesis was determined using [^3^H]-thymidine incorporation assay. (**B**) Scratch wounds were established in PCCs cultured to confluence. Images of the wound area were taken immediately and 24 h after incubation with SFM or PSC-CM. The wound area was measured using FIJI software. Data are mean ± SEM of triplicate (**A**) and 6–12 measurements (**B**). * *p* < 0.05, ** *p* < 0.01 comparing SFM (control) vs PSC-CM. (**C**,**D**) Heatmap showing inter-tumor variability of protein abundance for selected proteins that are proposed to regulate migration of pancreatic cancer cells. Low (green) to high (red) expression pattern was drawn for each individual protein for both PSCs (**C**) and PCCs (**D**). PCC, pancreatic cancer cell; PSC, pancreatic stellate cell; PSC-CM, PSC-conditioned medium; SFM, serum-free DMEM.

Preliminary studies revealed that prolonged incubation of PCCs with PSC-CM in itself were harmful, as it resulted in detachment of the cells (data not shown). Growth studies revealed that PCCs require a minimum of 1% FBS in the culture medium for normal adherence and growth. Thus, all experiments involving PSC-CM were carried out with PSC-CM containing 1% FBS. To evaluate the effect of PSC-CM on the proliferation of PCCs, PCC-2, -3, -4, -5, and -6 were incubated for 24 h with SFM or PSC-CM from paired PSCs followed by [^3^H]-thymidine incorporation for measurement of DNA synthesis (Figure 5A). Although statistically significant differences were not observed, each PSC-CM demonstrated a variable effect on DNA synthesis of the paired PCC: reduced in PCC-2 (*p* = 0.06), increased in PCC-3 (*p* = 0.06), and, interestingly, exhibited no change in NAT-PCCs (Figure 5A). Next, the migration capacity of PCCs upon exposure to SFM or PSC-CM was evaluated using the scratch wound assay. Compared to SFM, PSC-CM induced significantly higher migration of PCC-2, -3, -5, and -6. The observed increase in wound closure induced by PSC-CM was 350, 170, 360, and 200 percent for PCC-2, -3. -5, and -6, respectively (Figure 5B, Supplementary Materials Figure S5). The secretome data from the five paired PCCs and PSCs was further examined for proteins deemed to be involved in the regulation of migration of cancer cells [25,41,42]. Secretome analysis revealed significant intertumoral variability with respect to protein secretion in both PSCs and PCCs, as indicated in Figure 5C,D. Interestingly, most of these proteins were secreted in large amounts by PSC-2 and PSC-5, whereas the secretomes from PCC-3 and PCC-4 displayed significantly lower amounts of various integrins, ezrin, and MET, factors that are known to induce the activation of various signaling pathways leading to migration of cancer cells (Figure 5C,D). These findings could possibly explain the observed variation in PSC-induced migration of their paired PCCs. 

## 4. Discussion

The study of the cellular interactions taking place between the cancer and stroma has recently emerged as an important topic in pancreatic cancer research [43,44,45]. Multiple new stromal targets of potential clinical importance in PDAC are currently being investigated [46]. Of these, the activated PSCs, also denoted cancer-associated fibroblasts, have gained particular interest [47]. The majority of preclinical in vitro studies on the interactions between epithelial and stromal components of PDAC have been performed using a set of widely available cancer cells and PSCs obtained from diverse sources not necessarily representing the initial tumor conditions. In the present study, we aimed to develop a cellular model system consisting of primary PCCs and primary PSCs that were isolated from the same human PDAC. To the best of our knowledge, the present study is the first report on the establishment and initial characterization of human PDAC-derived pairs of primary PCCs and PSCs isolated from the same tumor specimen.

The six source tumors were histologically confirmed PDACs, including three treatment-naïve tumors and three neoadjuvantly treated tumors. Morphological assessment confirmed an epithelial appearance of all PCCs, and immunostaining for various markers identified their cytogenic characteristics. Expression of CK19 in all PCC cultures confirmed their ductal origin [48]. Both the mesenchymal marker vimentin and the epithelial marker EpCAM are known to be overexpressed in most pancreatic carcinomas [49,50]. Interestingly, PCC-3 and PCC-4 expressed vimentin but were negative for EpCAM. Increased expression of vimentin has been reported in neoplastic transformation and in vitro cultures [48]. Overall, proliferative activity as analyzed by Ki-67 expression was high though variable between the six PCC cultures. A high Ki-67 index has been reported in commercially available pancreatic cancer cell lines [51]; however, the average ki-67 index was unexpectedly higher across PCCs that is possibly related to the in vitro culture adaptation. CD44, a marker for cancer stem cells, is involved in many different cellular mechanisms; however, the different functional roles of CD44 standard and its isoforms are not fully understood. Upregulation of CD44 is correlated with tumor progression and the metastatic phenotype in many cancers, including pancreatic cancer [52]. All six PCC cultures demonstrated a strong expression of pan-CD44, consistent with previously reported data in human PDAC-derived carcinoma cells [53].

More than 90% of pancreatic cancers contain a mutated *KRAS* gene [54]. In the present study, all six PCC cultures harbored mutations in codon 12 or 13 of the *KRAS* gene, consistent with the importance of *KRAS* in PDAC carcinogenesis. Mutations of the tumor suppressor gene *CDKN2A* are reported in 49–98% of PDACs. *CDKN2A* encodes for p16^INK4^ and p14^ARF^, both proteins essential for cell-cycle regulation [55,56]. *CDKN2A* mutations were detected in three out of the six PCCs, affecting the reading frames of both proteins. Mutations of the tumor suppressor genes *TP53* and *SMAD4*/*DPC4* are observed in 20–76% and 19–50% of PDACs, respectively [55,56]. *TP53* is essential for stabilization of the cellular stress response, whereas *SMAD4* is involved in downstream signaling of the transforming growth factor *β* (TGF-*β*) receptor [56]. *SMAD4* function is known to be lost in 50% of PDACs, and its expression correlates with the differentiation of the carcinoma [57]. Mutations in *TP53,* believed to be somatic, were detected in three of the PCC cultures, of which two are categorized as damaging, whereas the third is of unknown significance. *SMAD4* tested negative for exon-specific mutations in all cell lines. Germline mutations of the tumor suppressor gene *BRCA2* are associated with a 3.5-fold increased risk of pancreatic cancer, and the gene is identified as the most common inheritable cause [58]. Four out of the six PCC cultures harbored mutations in the *BRCA2* gene. The main purpose of the genotyping was to assess the genetic heterogeneity among the cell cultures. Based on the combined detected aberrations in *KRAS*, *TP53*, and *CDKN2A*, all six cell cultures had distinguishable genotypes. However, the mutational status of the four genes also including the *SMAD4* gene does not allow apparent correlations to be made either to the grade of PDAC differentiation or to biological behavior [59].

PDAC is characterized by an exceedingly prominent stroma. PSCs are the main cellular component of the stroma, and they are known to interact with the cancer cells and to affect their behavior [11,19]. In a recent study, we demonstrated that primary PSCs and commonly used PSC-cultures differ phenotypically and also in their interactions with pancreatic cancer cells, which highlights the importance of selecting appropriate PSC cultures for a particular experiment [25]. All PSCs established in the present study strongly expressed the mesenchymal marker vimentin and the activation marker α-SMA consistent with the known characteristics of PSCs. Cell growth data revealed that the PSCs were growing at a much slower rate, compared to PCCs, with an average 3-fold higher doubling time. Interestingly, NAT-PCCs demonstrated an average 2-fold lower proliferation rate compared to TN-PCCs, suggesting that prior treatment with chemotherapeutic agents may have reduced overall proliferation capacity or the number of proliferating cells among NAT-PCCs. This notion was further supported by the findings from measurements of the percentage of Ki-67 positive cells (Ki-67 index), which revealed a trend towards lower Ki-67 index in NAT-PCCs (*p* = 0.06) compared to TN-PCCs.

Several recent reports have studied the complex interactions between PSCs and cancer cells based on analysis of their secretomes [42,60,61]. This prompted us to investigate the composition of the secretome from the paired primary PCCs and PSCs by proteomics-based analysis. The secretome composition confirmed the distinctly different nature of the cultures as stromal and epithelial. Of a total of 3209 proteins identified, 1301 proteins were differentially expressed between PCCs and PSCs. The PSC-CM expressed large amounts of various ECM components, including collagens, fibronectin, and SPRAC, whereas PCC-CM predominantly expressed proteins related to cell growth and to transcription and translation processes. Notably, PCC-CM displayed a significantly higher expression of HSP90AA1, a stress-inducible isoform of heat shock protein that has been shown to correlate with unfavorable prognosis of breast cancer patients [62]. Furthermore, the PSC secretomes are known to promote cancer cell proliferation and migration [25,40]. However, the majority of these studies were conducted using PSCs and PCCs that originated from different sources. In this study, we analyzed the ability of PSC-CM to influence proliferation and migration of the paired PCCs isolated from the same tumor. Although PSC-CM differentially affected proliferation of TN-PCCs; overall, no significant change in PSC-CM-induced proliferation of PCCs was observed. All five PSC secretomes induced the migration of the paired PCCs, albeit to a variable degree. The high variability in migration induction by the various PSCs may reflect intertumoral heterogeneity. Several proteins secreted by PSCs including cytokines, growth factors, and ECM components are known to interact with the PCCs and to induce activation of various signaling pathways that lead to enhanced migration and proliferation of the cancer cells [19,25,41,42,63]. The secretome data from both PSCs and PCCs investigated for these proteins revealed that the extent of migration observed among various PCCs was directly proportional to the amount of ligands/substrate proteins secreted by PSCs as well as to the amount of receptor proteins from PCCs. It is noteworthy that none of the five PSCs displayed detectable levels of hepatocyte growth factor (HGF), which is reported to be secreted in variable amounts by activated PSCs [40]. These findings could potentially explain the observed inter-tumor heterogeneity and also highlights the challenges in identifying therapeutic targets. 

Of note, there are certain limitations of the present study. Firstly, there is only a small number of patients (i.e., *n* = 3) in each group of treatment naïve and neoadjuvantly treated source tumors from which paired cell cultures were successfully obtained. Secondly, the conditioned medium was obtained from PCC and PSC cultured individually, which may not necessarily mimic real in vivo circumstances. At present, a further limitation is the lack of data indicating the use of these cells for the tumor formation in vivo using mouse models. 

In conclusion, this is the first study to demonstrate the successful establishment of human PDAC-derived paired primary cultures of PCCs and PSCs that are isolated from the same tumor specimen. Both PCC and PSC cultures show immunohistological and molecular properties that are characteristic for the respective cell types. The combination of these paired cultures of PCCs and PSCs enables the development of a novel cellular system with the potential to serve as a useful experimental model for investigations of the biology of PDAC as well as its response to treatment.

## Figures and Tables

**Table 1:** Clinicopathological features of source pancreatic adenocarcinoma (PADC) tumors.

PDAC ID	Tumor Site	TN-Stage	Tumor Size (mm)	Grade	Treatment	TRG	L	V	Pn	R	Survival (Months)
PC-1	HOP	pT2(3)N0	35 × 28 × 25	2	NA	NA	0	0	0	0	19.3
PC-2	HOP	pT2(3) N2(1)	39 × 35 × 30	2	NA	NA	1	1	1	1	10.3
PC-3	HOP	pT2(3) N1	26 × 24 × 21	3	NA	NA	1	1	1	0	11.9
PC-4	HOP	ypT3 N1	42 × 35 × 31	NA	4× Folfirinox	3	1	0	1	1	33.0+
PC-5	HOP	ypT3 N1	55 × 21 × 10	NA	1× Folfirinox, 5× Gemzar -Abraxane	2–3	1	1	1	1	11.5
PC-6	HOP	ypT3N2(1)	52 × 50 × 32	NA	7× Gemzar	3	1	0	1	1	12.8

One biopsy per tumor from six individual PDACs (PC-1 to PC-6) were used to isolated paired cultures of pancreatic cancer cells (PCCs) and pancreatic stellate cells (PSCs). pTN-staging according to UICC TNM 8. Edition (from 2018). In brackets are the stage according to 7th edition if different from 8th edition. PC, pancreatic cancer; HOP, head of pancreas; NA, not applicable: TRG, tumor regression grade (according to CAP = College of American Pathologists).

**Table 2:** Immunocytochemical analysis of markers for PDAC-derived primary PCC cultures.

Target	PCC-1	PCC-2	PCC-3	PCC-4	PCC-5	PCC-6	BxPC-3
CK19	++	++	++	++	++	++	++
EpCAM	++	++	-	-	++	++	-
Vimentin	-	-	++	++	-	-	-
Ki-67	++	++	++	+	+	+	-
p53	+	+	++	++	+	+	+
SMAD4	++	++	++	+	+	-	-
CD44 (ic)	+	+	+	+	+	+	-
Caspase-3	++	++	+	+	+	+	+
p16	++	++	+	+	+	++	-

BxPC-3 was used as positive control. PCC, pancreatic cancer cell; ic, immunocytochemistry; -, low or no detectable expression, +, partial/low expression, ++, strong expression.

**Table 3:** Mutation profiles of PDAC-derived primary PCC cultures with allele fraction (AF) values.

Target (RefSeq)	PCC-1	PCC-2	PCC-3	PCC-4	PCC-5	PCC-6	AsPC-1	Panc-1
*KRAS* (nm_004985)	p.G13D (0.47)	p.G13D (0.46)	p.G12C (1.0)	p.G12C (1.0)	p.G12C (0.78)	p.G12V (0.67)	p.G12D (0.99)	p.G12C (0.49)
*TP53* (nm-000546)	p.K373fs (0.33)	-	-	p.D21E (0.21)	-	p.A138V (1.0)	p.C135fs (0.86)	-
*CDKN2A* (nm_000077)	p.G23fs (0.37) p.E33fs (0.45)	p.G23fs (0.35) p.E33fs (0.40)	-	-	p.G23fs (0.35) p.E33fs (0.40)	-	p.L78fs (0.77)	p.G23fs (0.35) p.E33fs (0.40)
*SMAD4* (nm_005359)	-	-	-	-	-	-	p.R100T (1.0)	-
*BRCA1* (nm_007294)	-	-	-	-	-	-	p.D693N (1.0)	-
*BRCA2* (nm_000059)	p.I2672fs (0.32)	p.I2672fs (0.37)	-	-	p.I2672fs (0.20)	p.N372H (0.54)		p.I2672fs (0.32)

DNA from AsPC-1 and Panc-1 cells were used as controls. PCC, pancreatic cancer cell; -, not detected.

## Supplementary Materials

The following are available online at https://www.mdpi.com/2073-4409/9/1/227/s1. Figure S1: Schematic representation of the procedure for establishment PCCs and PSCs, Figure S2: Analysis of secretome from PCCs and PSCs, Figure S3: Immunostaining of PCCs, Figure S4: Comparing secretomes of treatment naïve and neoadjuvantly treated PCCs and PSCs; Figure S5: Effect of PSC-CM on cancer cell migration. Table S1: Antibodies, Table S2: Secretome data, Table S3: Analysis of secretome data.
