# Supplementary material for: Establishment and Characterization of Paired Primary Cultures of Human Pancreatic Cancer Cells and Stellate Cells Derived from the Same Tumor
Source: Cells. 2020 Jan 16;9(1):227. doi: 10.3390/cells9010227 (PMC7016771; doi:10.3390/cells9010227)
Supplement: Supplementary file 1 [file cells-09-00227-s001.zip › Supplementary Material/Supplementary Material Table S1.pdf]

Table S1. Antibodies

Name	Company	Cat.no.	Dilution	Application
Primary antibodies				
Cytokeratin 19	Abcam	ab52625	1:200<br>1:1000	ICC<br>WB
EpCAM	Cell Signaling Technology	#2929	1:50<br>1:1000	ICC<br>WB
Vimentin	Cell Signaling Technology	#5741	1:200<br>1:1000	ICC<br>WB
Ki-67	Dako	M7240	1:200<br>1:1000	ICC<br>WB
CD44	Dako	M7082	1:200<br>1:1000	ICC<br>WB
Caspase-3	Cell Signaling Technology	#9662	1:200<br>1:1000	ICC<br>WB
p16	Santa Cruz Biotechnology Inc.	sc-467	1:200	ICC
p53	Cell Signaling Technology	#2524S	1:200<br>1:1000	ICC<br>WB
SMAD-4	Cell Signaling Technology	D3M6U	1:1000	WB
α-SMA	Nordic Biosite AB	BSH-7459	1:200<br>1:1000	ICC<br>WB
Secondary antibodies (IgG)				
Alexa Fluor 488 AffiniPure Donkey Anti-Mouse	Jackson ImmunoResearch Laboratories, Inc.	715-545-150	1:500	ICC
Alexa Fluor 594 AffiniPure Goat Anti-Rabbit	Jackson ImmunoResearch Laboratories, Inc.	111-585-144	1:500	ICC
HRP-Conjugated Goat Anti-Mouse	Bio-Rad Laboratories	1706516	1:10 000	WB
HRP-Conjugated Goat Anti-Rabbit	Bio-Rad Laboratories	1706515	1:10 000	WB

h, human PDAC tissue; ICC, immunocytochemistry (immunostaining); IHC, immunohistochemistry; WB, western blot (immunoblotting)
