# Supplementary material for: Establishment and Characterization of Paired Primary Cultures of Human Pancreatic Cancer Cells and Stellate Cells Derived from the Same Tumor
Source: Cells. 2020 Jan 16;9(1):227. doi: 10.3390/cells9010227 (PMC7016771; doi:10.3390/cells9010227)
Supplement: Supplementary file 1 [file cells-09-00227-s001.zip › Supplementary Material/Supplementary Material Figure S1.pdf]

Figure S1

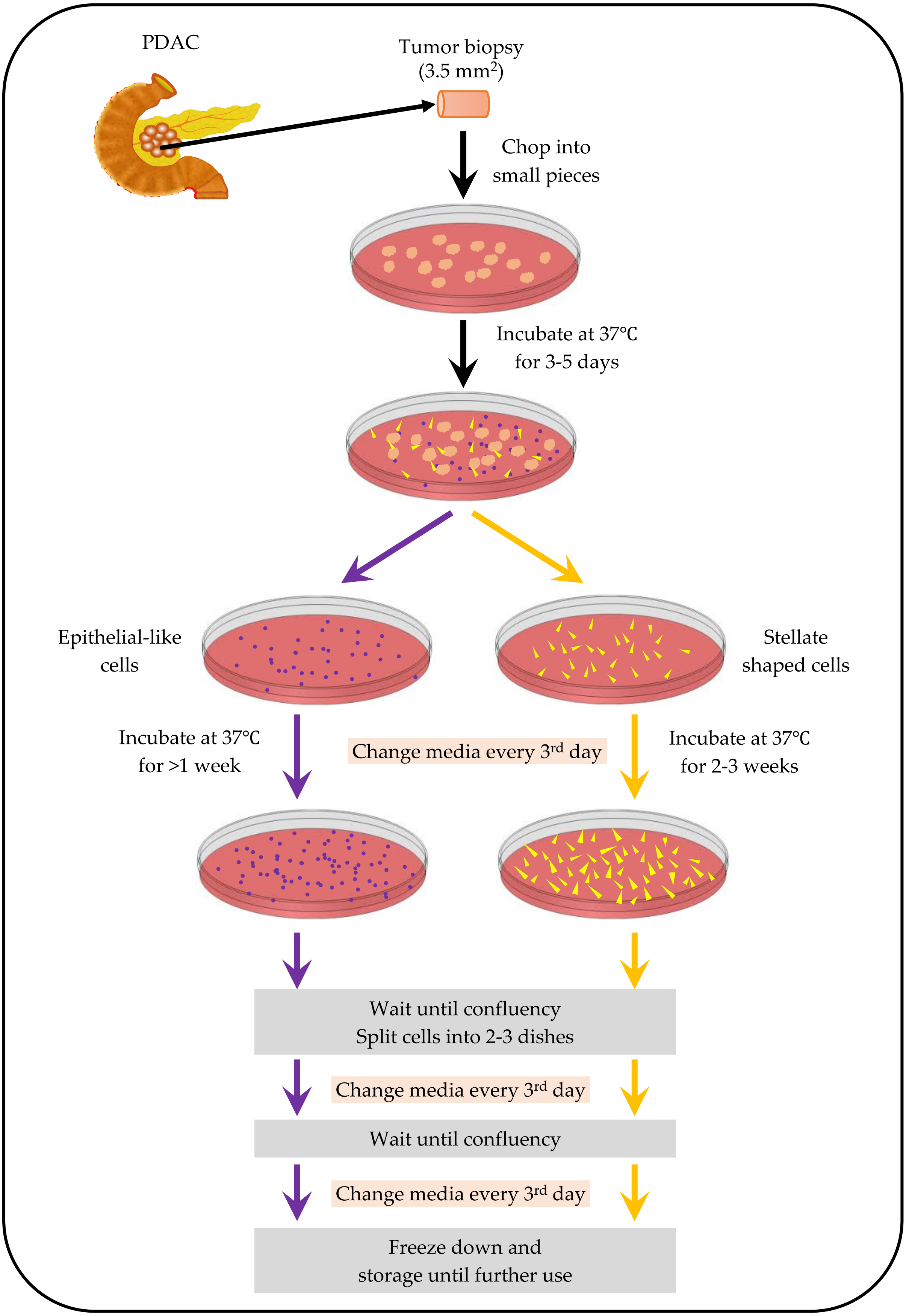

Supplementary Material Figure S1. Schematic representation of the procedure for establishment of human PDAC-derived primary cultures of pancreatic cancer cells (PCCs) and pancreatic stellate cells (PSCs) by outgrowth method. PDAC, pancreatic ductal adenocarcinoma.
