# Supplementary material for: Establishment and Characterization of Paired Primary Cultures of Human Pancreatic Cancer Cells and Stellate Cells Derived from the Same Tumor
Source: Cells. 2020 Jan 16;9(1):227. doi: 10.3390/cells9010227 (PMC7016771; doi:10.3390/cells9010227)
Supplement: Supplementary file 1 [file cells-09-00227-s001.zip › Supplementary Material/Supplementary Material Figure S3.pdf]

# Figure S3

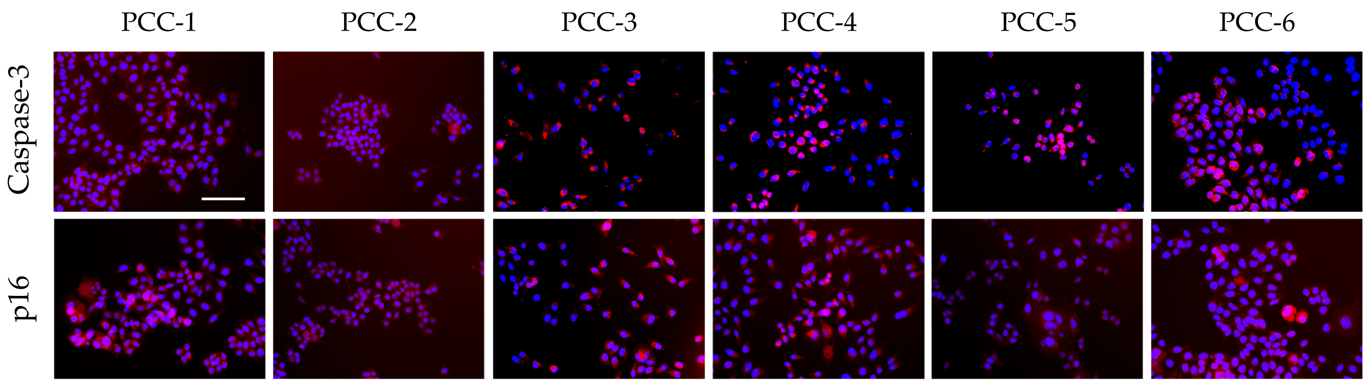


**Supplementary Material Figure S3.** PCCs immunostained with antibodies against caspase-3 and p16 (red). Nuclei were stained with DAPI (blue). Scale bar= 100 μM. PCC, pancreatic cancer cell.
