# Supplementary material for: Establishment and Characterization of Paired Primary Cultures of Human Pancreatic Cancer Cells and Stellate Cells Derived from the Same Tumor
Source: Cells. 2020 Jan 16;9(1):227. doi: 10.3390/cells9010227 (PMC7016771; doi:10.3390/cells9010227)
Supplement: Supplementary file 1 [file cells-09-00227-s001.zip › Supplementary Material/Supplementary Material Figure S5.pdf]

Figure S5

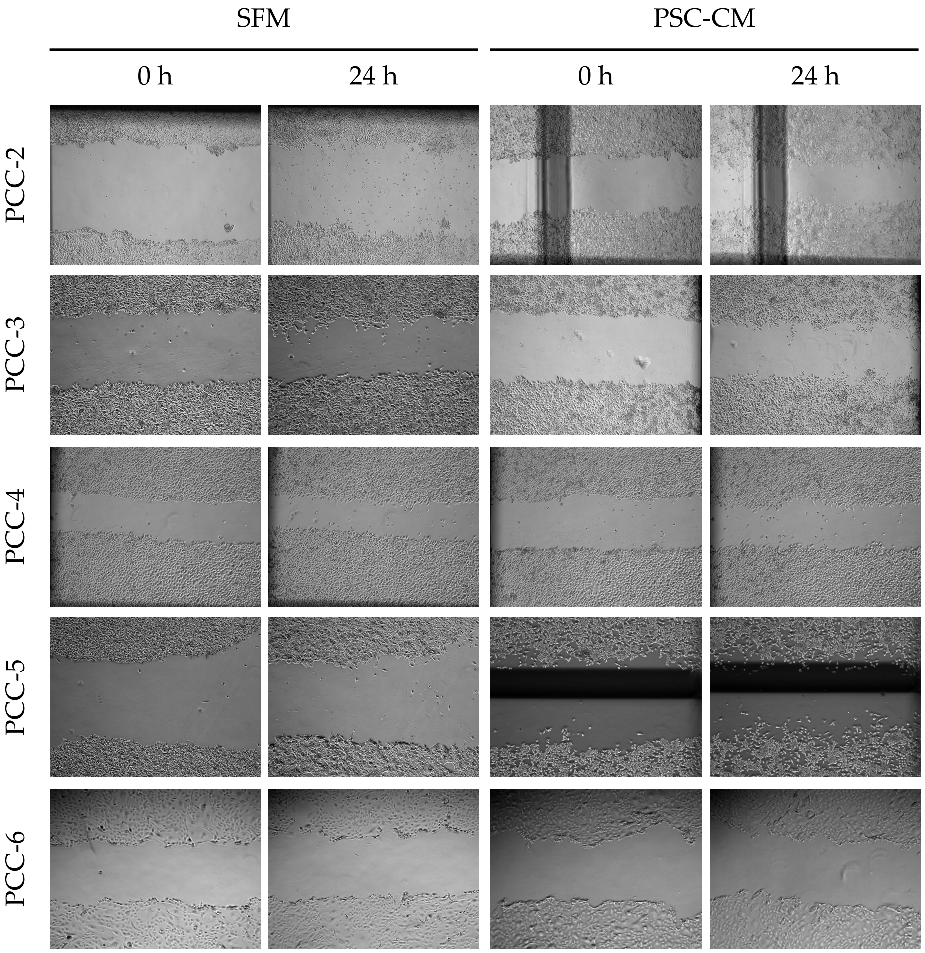

Supplementary Material Figure S5. Effect of PSC-CM on cancer cell migration. PDAC-derived primary cultures of pancreatic cancer cells (PCC-2, -3, -4, -5 and -6) were cultured to confluence and scratch wounds were established. The images of the wound area were taken immediately after the scratches and 24 h after incubation with SFM or respective paired PSC-CM. PSC, pancreatic stellate cell; PSC-CM, PSC conditioned medium; SFM, serum-free DMEM.
