# Supplementary material for: Establishment and Characterization of Paired Primary Cultures of Human Pancreatic Cancer Cells and Stellate Cells Derived from the Same Tumor
Source: Cells. 2020 Jan 16;9(1):227. doi: 10.3390/cells9010227 (PMC7016771; doi:10.3390/cells9010227)
Supplement: Supplementary file 1 [file cells-09-00227-s001.zip › Supplementary Material/Supplementary Material Figure S4.pdf]

Figure S4

A

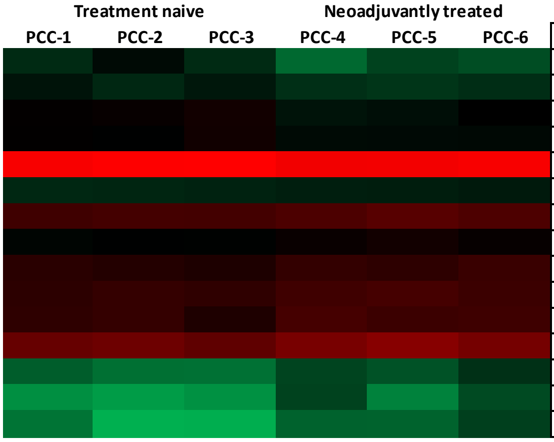

Protein name	Known functions
NAGLU	Glycosaminoglycan metabolism, degradation of heparan sulfate
PUS7	Protein synthesis
HNRNPUL2	Protein coding, Kinase activity
SPR	Metabolism, eNOS activation and regulation
PPIA	Peptide folding, formation of infectious HIV viruses
DR1	DNA binding and transcription
SERPINH1	Degradation of ECM
TUBA1A;TUBA3E	Microtubule assembly
UBA2	Ubiquitin mediated proteolysis
RBBP7	Cellular Senescence and regulation of TP53 activity
TPT1	Regulator of cellular growth and proliferation
HSP90B1	Molecular chaperone, processing and transport of secreted proteins
ANTXR1	Cell attachment and migration
COL4A1	Inhibits angiogenesis and tumor formation
COMP	Interacts with ECM proteins FN and Collagen, apoptosis supressor

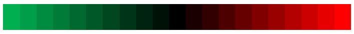

B

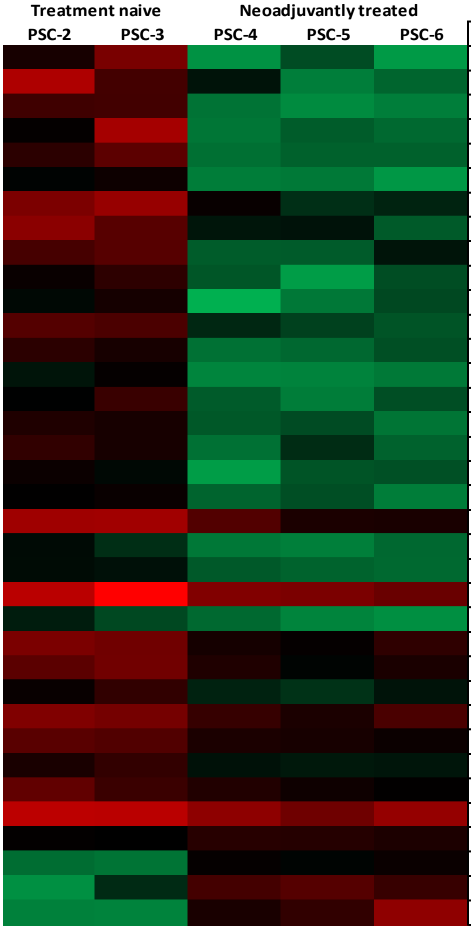

Protein name	Known functions
EIF1AX;EIF1AY	Protein biosynthesis
DYNLL1	Intracellular transport and motility
TUBB6	Microtuble assembly
SNRNP200	RNA splisosome
PNPO	Synthesis of pyridoxal 5'-phosphate (vitamin B6)
SEC24C	Vesicle trafficking
PTBP1	Pre-mRNA splicing
LTBP3	Integrin-dependent activation of TGF-beta
STX12	Protein transport between late endosomes and trans-Golgi network
NUDT21	Activator of the pre-mRNA 3'-end cleavag
GSPT1	Regulation of mammalian cell growth
NUDC	Necessary for cytokinesis and cell proliferation
RAC1;RAC3	Cell growth, cytoskeletal reorganization, activation of protein kinases
TIPRL	DNA damage response, DNA replication and repair
DHX15	Spliceosome disassembly
BIN1	Plasma membrane remodeling, MYC activity regulation and cell proliferation
CARM1	DNA packaging, transcription regulation
DPY30	Methylation of histone
MARS	Protein biosynthesis
PALLD	Organization of normal actin cytoskeleton, cell morphology
PCNA	DNA replication and DNA damage response
SNRPD3	Splisosomal regulation
STAM	Cytokines and growth factors mediated intracellular signal transduction
CBX3	Transcriptional silencing
SNX12	Intracellular trafficking
NFASC	Cell adhesion
RUVBL1	Transcriptional activation
PAFAH1B2	Inactivation of platelet-activating factor
PA2G4	Growth regulation
MGAT5	Regulation of the biosynthesis of glycoprotein oligosaccharides
RPS16	Ribosomal protein
B4GALT1	Cell cell and cell matrix interactions
GALNS	Degradation of the glycosaminoglycans
CAPN1	Cytoskeletal remodeling and signal transduction
ERP44	Chaperone of the secretory pathway
CGREF1	Cell-cell adhesion

Supplementary Material Figure S4. Secretome analysis of conditioned medium from PCCs and PSCs. Conditioned medium from six different PCC and five PSC cultures, were subjected to proteomics analysis using LC-MS/MS. Heatmap showing a list of protein abundance pattern of differentially regulated proteins comparing between PCCs (A) and PSCs (B) derived from treatment naïve and neoadjuvantly treated tumors. The table indicates protein names and their known functions according to GeneCards®: The Human Gene Database (https://www.genecards.org/). Red and green color indicates high and low expression, respectively. PCC, pancreatic cancer cell; PSC, pancreatic stellate cell.
