# Supplementary figures and images for: Establishment and Characterization of Paired Primary Cultures of Human Pancreatic Cancer Cells and Stellate Cells Derived from the Same Tumor
Source: Cells. 2020 Jan 16;9(1):227. doi: 10.3390/cells9010227 (PMC7016771; doi:10.3390/cells9010227)

Figure S2

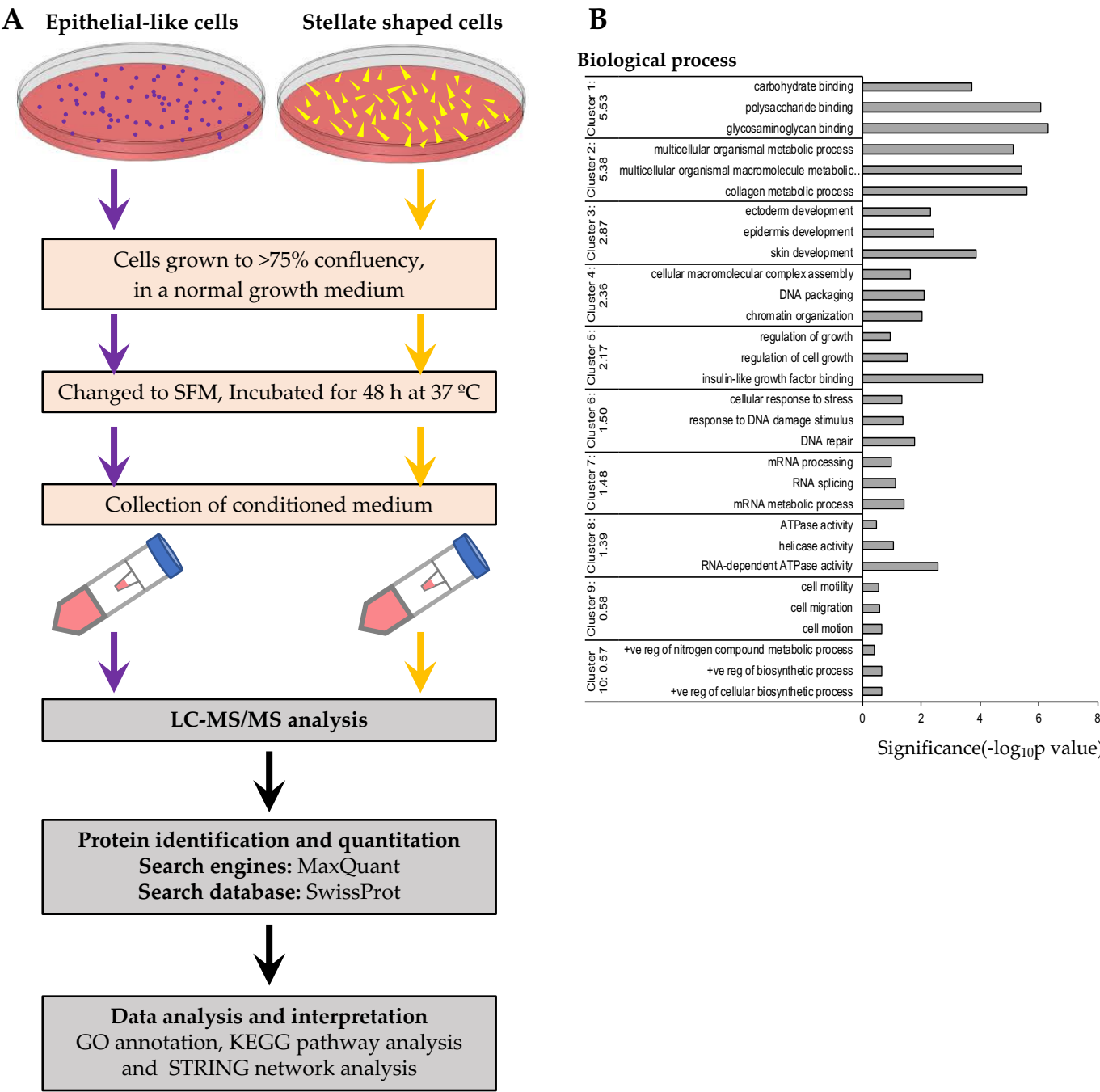

Supplement: Supplementary file 1 [file cells-09-00227-s001.zip › Supplementary Material/Supplementary Material Figure S2.pdf]
